# Evaluation of the Efficacy of the Brucella canis RM6/66 Δ*vjbR* Vaccine Candidate for Protection against B. canis Infection in Mice

**DOI:** 10.1128/mSphere.00172-20

**Published:** 2020-05-20

**Authors:** Lauren W. Stranahan, Sankar P. Chaki, Daniel G. Garcia-Gonzalez, Omar H. Khalaf, Angela M. Arenas-Gamboa

**Affiliations:** a Department of Veterinary Pathobiology, College of Veterinary Medicine, Texas A&M University, College Station, Texas, USA; b Department of Veterinary Pathology & Poultry Diseases, College of Veterinary Medicine, University of Baghdad, Baghdad, Iraq; University of Florida

**Keywords:** *Brucella canis*, brucellosis, modified live vaccine, mouse model, vaccine, vaccine efficacy, vaccine safety

## Abstract

Brucella canis is a Gram-negative, facultative intracellular bacterium and the causative agent of canine brucellosis, a highly contagious disease of dogs that can be transmitted to humans. Unfortunately, no vaccine is available to prevent infection. We recently characterized the kinetics of B. canis infection in the mouse model, establishing the required dose necessary to achieve systemic infection. The objective of this study was to investigate the utility of the mouse model in assessing canine brucellosis vaccine candidates and to subsequently investigate the safety and efficacy of a live attenuated vaccine, the B. canis RM6/66 Δ*vjbR* strain. Mice vaccinated with a dose of 10^9^ CFU of the vaccine strain by both intraperitoneal and subcutaneous routes were afforded significant protection against organ colonization and development of histopathologic lesions following intraperitoneal challenge. Addition of an adjuvant or a booster dose 2 weeks following initial vaccination did not alter protection levels. Vaccination also resulted in a robust humoral immune response in mice, and B. canis RM6/66 Δ*vjbR* was capable of activating canine dendritic cells *in vitro*. These data demonstrate that the B. canis RM6/66 Δ*vjbR* strain shows promise as a vaccine for canine brucellosis and validates the mouse model for future vaccine efficacy studies.

**IMPORTANCE** Canine brucellosis, caused by Brucella canis, is the primary cause of reproductive failure in dogs and represents a public health concern due to its zoonotic nature. Cases in dogs in the United States have been increasing due to the persistent nature of the bacterium, deficiencies in current diagnostic testing, and, most importantly, the lack of a protective vaccine. Current estimates place the seroprevalence of B. canis in the southern United States at 7% to 8%, but with the unprecedented rates of animals moving across state and international borders and the lack of federal regulations in regard to testing, the true seroprevalence of B. canis in the United States may very well be higher. Vaccination represents the most effective method of brucellosis control and, in response to the demand for a vaccine against B. canis, we have developed the live attenuated B. canis RM6/66 Δ*vjbR* vaccine strain capable of protecting mice against challenge.

## Abstract (precis)

Canine brucellosis, caused by Brucella canis, is the primary cause of reproductive failure in dogs and represents a public health concern due to its zoonotic nature. Cases in dogs in the United States have been increasing due to the persistent nature of the bacterium, deficiencies in current diagnostic testing, and, most importantly, the lack of a protective vaccine. Current estimates place the seroprevalence of B. canis in the southern United States at 7% to 8%, but with the unprecedented rates of animals moving across state and international borders and the lack of federal regulations in regard to testing, the true seroprevalence of B. canis in the United States may very well be higher. Vaccination represents the most effective method of brucellosis control and, in response to the demand for a vaccine against B. canis, we have developed the live attenuated B. canis RM6/66 Δ*vjbR* vaccine strain capable of protecting mice against challenge.

## INTRODUCTION

*Brucella*, a facultative intracellular bacterium, is the causative agent of brucellosis, a devastating disease in multiple domestic and wild mammalian species and the most commonly reported zoonotic disease in humans worldwide (1). Brucella canis is the agent responsible for canine brucellosis, and although it most commonly infects dogs, it may also be transmitted to humans, in which it can cause a chronic febrile disease (2, 3). As with other *Brucella* species, B. canis has a tropism for the reproductive system and frequently causes abortion as well as epididymitis and prostatitis in male dogs (4, 5). In addition to reproductive manifestations, dogs may present with lymphadenopathy, recurrent uveitis, or diskospondylitis (2, 6). Alarmingly, infected dogs may present with nonspecific clinical signs or remain asymptomatic, resulting in missed diagnoses (2). This poses a significant health risk to other dogs as well as to humans, as silently infected dogs may continuously shed the bacteria for as long as 5.5 years, serving as an ongoing and undetected source of infection (7). Growing evidence suggests that the prevalence of canine brucellosis in the United States and other parts of the world is increasing, likely owing to a combination of the persistent nature of the pathogen, difficulties in diagnosing infected dogs, an absence of approved surveillance and prevention strategies in most countries, and most importantly, the lack of a protective vaccine (2, 8).

It has become clear over the past 50 years that control of brucellosis in any species is unachievable without vaccination (9). Due to safety concerns associated with currently available live attenuated vaccines (LAVs) in domestic ruminants, such as induction of abortion in pregnant animals and residual virulence for humans, research efforts into new vaccine development have expanded into DNA and subunit preparations (9). In recent years, the majority of investigated *Brucella* vaccine candidates fall within this category, with fewer studies investigating LAVs (10–15). However, LAVs consistently provide the greatest levels of protection in the context of brucellosis due to their ability to generate more persistent memory responses (16, 17).

To develop safer LAVs, our laboratory has generated several promising vaccine candidates through deletion of the *vjbR* gene, which encodes the LuxR-like quorum-sensing-related transcriptional regulator which is required for expression of the *virB* operon in both rough and smooth *Brucella* spp. and is critical for survival in phagocytes and virulence in mice (18, 19). Deletion mutants generated from Brucella abortus S19 and Brucella melitensis 16M have proven to confer significant levels of protection in the mouse model and to be safe in natural swine and ruminant hosts (20–23).

Mice have become the most frequently utilized animal model for investigating candidate vaccines for brucellosis due, in large part, to substantial economic and ethical constraints associated with studies involving natural hosts, such as dogs (24). While results obtained from mice are not directly transferable to humans or natural animal hosts, important information regarding safety and the potential for induction of protective cell-mediated immunity by candidate vaccines can be readily attained using the mouse model (24). We recently characterized the kinetics and pathological lesions induced by B. canis in mice and determined an appropriate challenge dose for use in future canine brucellosis vaccine studies (25). In this study, we utilized this model to investigate the safety and protective efficacy of the B. canis RM6/66 Δ*vjbR* vaccine candidate. Additionally, the effects of vaccine route and formulation were interrogated as well as the potential for the vaccine to activate dendritic cells in dogs, providing further evidence to support conduction of safety and efficacy studies in the natural host.

## RESULTS

### The B. canis RM6/66 Δ*vjbR* strain is attenuated in cells and mice.

**FIG 1:**
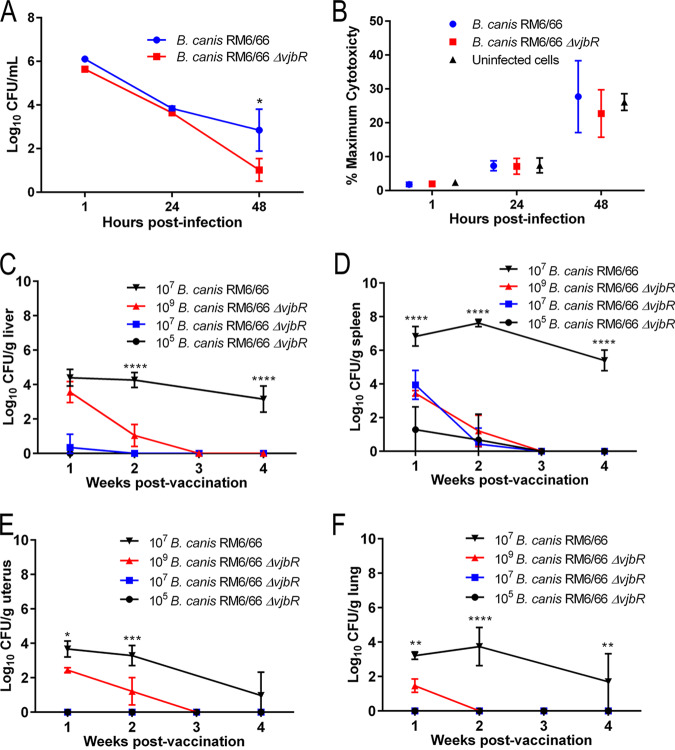
B. canis RM6/66 Δ*vjbR* is attenuated in canine cells and in C57BL/6 mice. (A) Canine DH82 macrophage-like cells were infected with B. canis RM6/66 Δ*vjbR* and wild-type B. canis RM6/66 at MOI of 100, and the number of intracellular bacteria was quantified at 1, 24, and 48 h postinfection. (B) Assessment of induction of cell death was determined via measurement of lactate dehydrogenase (LDH) in cell culture supernatants at 1, 24, and 48 h postinfection. Female C57BL/6 mice (*n* = 5) were infected i.p. with various doses of B. canis RM6/66 Δ*vjbR* or 10^7^ CFU of B. canis RM6/66, and bacterial colonization in the liver (C), spleen (D), uterus (E), and lung (F) was monitored over 4 weeks. Data are presented as means ± standard deviations. *, *P* < 0.05; **, *P* < 0.01; ***, *P* < 0.001; ****, *P* < 0.0001 (Tukey’s multiple-comparison test).

The B. canis RM6/66 Δ*vjbR* vaccine strain was constructed by deletion of a 753-bp segment from the *vjbR* gene (BCAN_RS10670) and verified following PCR and sequencing (see Fig. S1 in the supplemental material). To assess the virulence of B. canis RM6/66 Δ*vjbR* in comparison to that of wild-type B. canis RM6/66, growth and survival were assessed *in vitro*. For the first time, a cell line derived from the natural canine host, DH82 macrophage-like cells, was used to evaluate a canine brucellosis vaccine candidate. B. canis RM6/66 Δ*vjbR* was capable of invading cells at a similar rate as wild-type B. canis, with no significant differences observed in bacterial numbers at 1 and 24 h postinfection. However, by 48 h, the B. canis RM6/66 Δ*vjbR* CFU were significantly reduced compared to that of the wild type, indicating attenuation of the vaccine strain *in vitro* (Fig. 1A). Impact of infection on cell viability was determined via measurement of lactate dehydrogenase (LDH) release in cell culture supernatants, which revealed no significant differences between uninfected cells and those infected with both wild-type B. canis RM6/66 and B. canis RM6/66 Δ*vjbR* (Fig. 1B).

10.1128/mSphere.00172-20.1 FIG S1 Confirmation and selection of B. canis RM6/66 Δ*vjbR* clones. PCR amplification shows a mixture of wild-type (WT), merodiploid, and mutant colonies. Clone 7 (C7) in lane 4 and clone 9 (C9) in lane 6 were selected as B. canis RM6/66 Δ*vjbR* mutants for use in vaccination studies. Successful production of B. canis RM6/66 Δ*vjbR* resulted in deletion of a 753-bp fragment, resulting in a final product size of 193 bp. Download FIG S1, TIF file, 0.5 MB. Copyright © 2020 Stranahan et al. 2020 Stranahan et al. This content is distributed under the terms of the Creative Commons Attribution 4.0 International license.

It is critical that candidate LAVs are cleared from the host within a reasonable time and do not induce persistent infection or signs of disease in the host. To assess the attenuation of B. canis RM6/66 Δ*vjbR in vivo* to this aim, dose titration studies were carried out in mice via intraperitoneal (i.p.) infection at various doses (Fig. 1). Mice were euthanized at weekly intervals, and colonization of organs for which *Brucella* spp. have a known tropism (liver, spleen, and uterus) was compared (1). Colonization of the lung was also assessed to provide an understanding of differences in systemic distribution. B. canis RM6/66 Δ*vjbR* colonized the spleen at all doses, while only higher doses of 10^7^ and 10^9^ CFU resulted in colonization of the liver (Fig. 1C and D). Additionally, the uterus and lung were only colonized by B. canis RM6/66 Δ*vjbR* at the high dose of 10^9^ CFU (Fig. 1E and F). For all doses of the vaccine strain, colonization was transient, and complete clearance was achieved in all examined organs and at all doses by 3 weeks postvaccination. The level of colonization in the liver by 10^9^ CFU B. canis RM6/66 Δ*vjbR* at 1 week postvaccination was similar to that achieved by wild-type B. canis at a dose of 10^7^ CFU. However, organ colonization at all doses and all remaining time points was significantly lower than with infection with 10^7^ CFU of wild-type B. canis. In contrast to B. canis RM6/66 Δ*vjbR*, mice infected with wild-type B. canis exhibited persistent colonization of all examined organs through 4 weeks postinfection, most notably in the spleen, with growth of nearly 5 logs at the 4-week time point. B. canis RM6/66 Δ*vjbR* is thus significantly attenuated both *in vitro* and *in vivo* in comparison to the parent strain.

### Vaccination with a high dose results in splenic extramedullary hematopoiesis.

**FIG 2:**
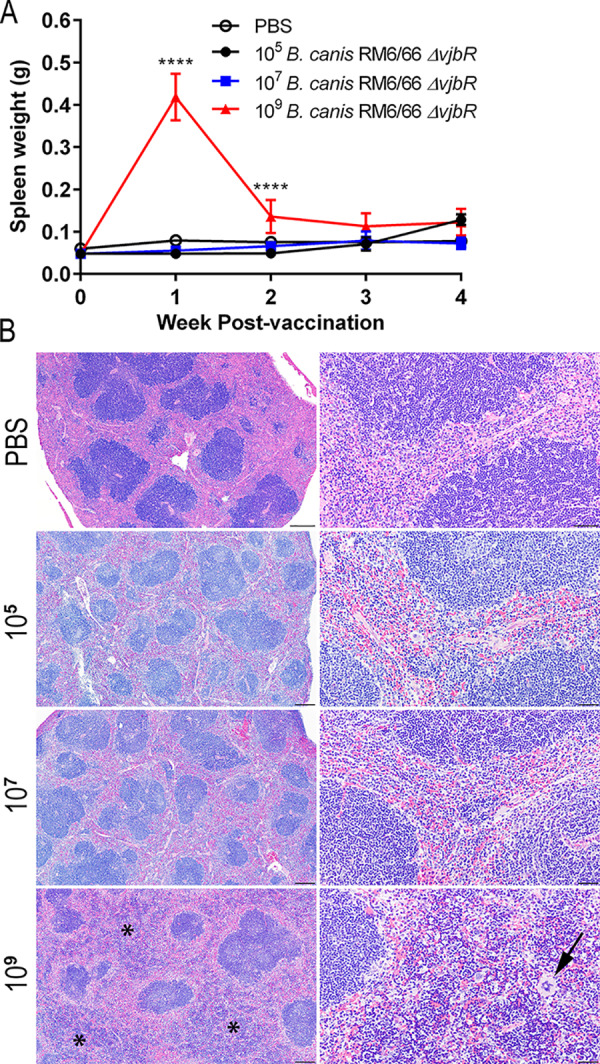
Vaccination of mice i.p. with 10^9^ CFU of B. canis RM6/66 Δ*vjbR* results in transient splenomegaly associated with extramedullary hematopoiesis (EMH). Mice were vaccinated i.p. with various doses CFU of B. canis RM6/66 Δ*vjbR* or PBS and euthanized at weekly intervals until 4 weeks postvaccination. (A) Splenic weight was determined in all groups at necropsy. Data are presented as means ± standard deviations. ****, *P* < 0.0001 (Tukey’s multiple-comparison test). (B) Hematoxylin and eosin (H&E) staining of the spleen at low magnification (left) and high magnification (right). Notice expansion of the red pulp sinuses (asterisks) by increased erythroid and myeloid precursor cells, as well as megakaryocytes (arrow), indicative of EMH in mice vaccinated with 10^9^ CFU. Bars, 200 μm (left) and 50 μm (right).

To further investigate the virulence and safety of B. canis RM6/66 Δ*vjbR* in mice, splenic weight was determined and organs were examined histologically at each weekly time point. At 1 week postvaccination, mice administered a high dose of 10^9^ CFU of B. canis RM6/66 Δ*vjbR* i.p. developed significant but transient splenomegaly that subsided by 3 weeks postvaccination (Fig. 2A). Histologically, spleens in mice vaccinated with the high dose exhibited marked expansion of the red pulp by megakaryocytes and erythroid and myeloid precursor cells, representing extramedullary hematopoiesis (EMH) (Fig. 2B). No histiocytic infiltration/granuloma formation was identified in the spleen in any of the vaccinated mice at any time point. Histologic examination of the liver demonstrated numerous foci of EMH within periportal areas at 2 weeks postvaccination in mice in the high-dose (10^9^ CFU) group. Mice in the high-dose group also exhibited scattered microgranulomas (70- to 150-μm diameter) composed of macrophages by 2 weeks postvaccination, a change not observed in the other dose groups (see Fig. S2). However, by 4 weeks postvaccination, the number of microgranulomas had markedly declined. No histologic lesions were observed in any other examined organ at any time point.

10.1128/mSphere.00172-20.2 FIG S2 Vaccination of mice with 10^9^ CFU of B. canis RM6/66 Δ*vjbR* results in histiocytic inflammation in the liver at 2 weeks postvaccination. Female C57BL/6 mice (*n* = 5) were vaccinated i.p. with 10^5^, 10^7^, or 10^9^ CFU of B. canis RM6/66 Δ*vjbR* or PBS and euthanized at weekly intervals through 4 weeks postvaccination. H&E staining of the liver at 2 weeks postvaccination at low magnification (left) and high magnification (right). Vaccination with a high dose of 10^9^ CFU resulted in the formation of multifocal microgranulomas (asterisks) as well as small foci of extramedullary hematopoiesis (EMH) (arrows) adjacent to portal and central veins. Bars, 200 μm (left) and 50 μm (right). Download FIG S2, PDF file, 0.7 MB. Copyright © 2020 Stranahan et al. 2020 Stranahan et al. This content is distributed under the terms of the Creative Commons Attribution 4.0 International license.

### Intraperitoneal vaccination with the B. canis RM6/66 Δ*vjbR* strain offers protection against wild-type challenge.

**FIG 3:**
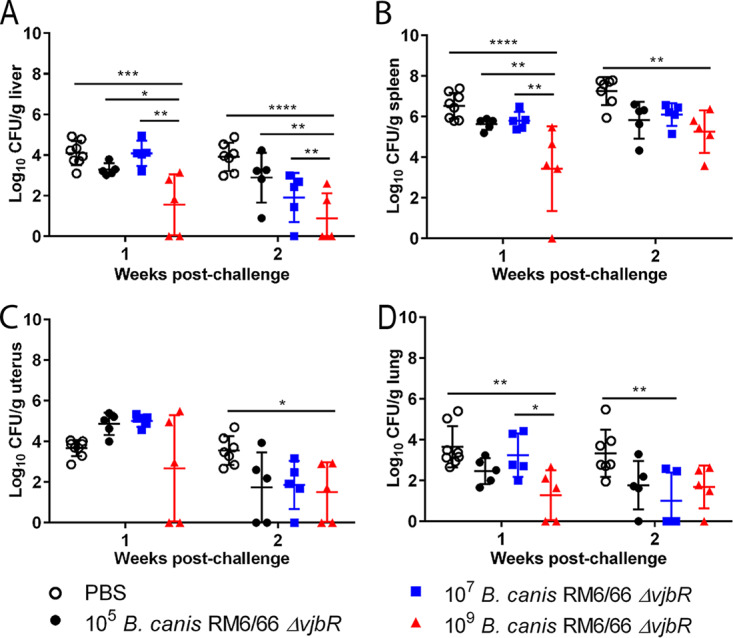
Vaccination with 10^9^ CFU of B. canis RM6/66 Δ*vjbR* results in transient protection against colonization following B. canis RM6/66 challenge. Female C57BL/6 mice (*n* = 5) were vaccinated i.p. with various doses of B. canis RM6/66 Δ*vjbR* or PBS, challenged i.p. at 4 weeks postvaccination with 10^7^ CFU of wild-type B. canis RM6/66, and euthanized at 1 and 2 weeks postchallenge. Organ colonization following challenge was assessed in the liver (A), spleen (B), uterus (C), and lung (D). All data are expressed as the means ± standard deviations. *, *P* < 0.05; **, *P* < 0.01; ***, *P* < 0.001; ****, *P* < 0.0001 (Tukey’s multiple-comparison test).

Following clearance of the vaccine strain by 3 weeks postvaccination, mice were challenged i.p. at 4 weeks postvaccination with a previously established challenge dose of 10^7^ CFU of B. canis RM6/66 (25). Organ colonization was assessed at 1 and 2 weeks postchallenge to determine protective efficacy. At 1 week postchallenge, mice vaccinated with the high dose of 10^9^ CFU of B. canis RM6/66 Δ*vjbR* exhibited significantly lower colonization in the spleen (3.092-log reduction), liver (2.543-log reduction), and lung (2.365-log reduction), with one mouse exhibiting no detectable colonization in the spleen and 2 mice demonstrating the same effect in the lung (Fig. 3). No significant reduction in uterine colonization was noted in the high-dose group at 1 week postchallenge, although 2 mice showed no colonization (Fig. 3C). Interestingly, the same 2 mice in the high-dose group exhibited no colonization in the liver, uterus, or lung. No reduction in organ colonization was noted in mice vaccinated i.p. with doses of 10^5^ or 10^7^ CFU (Fig. 3).

By 2 weeks postchallenge, again, mice vaccinated with the high dose of B. canis RM6/66 Δ*vjbR* exhibited significantly lower colonization in the spleen (2.0-log reduction) and liver (3.033-log reduction) (Fig. 3). Interestingly, mice vaccinated with the high dose of 10^9^ CFU demonstrated a 2.043-log reduction in uterine colonization at 2 weeks postchallenge. As opposed to that at 1 week postchallenge, the lung no longer displayed significant protection against colonization at 2 weeks postchallenge with the high-dose vaccination (Fig. 3D).

**FIG 4:**
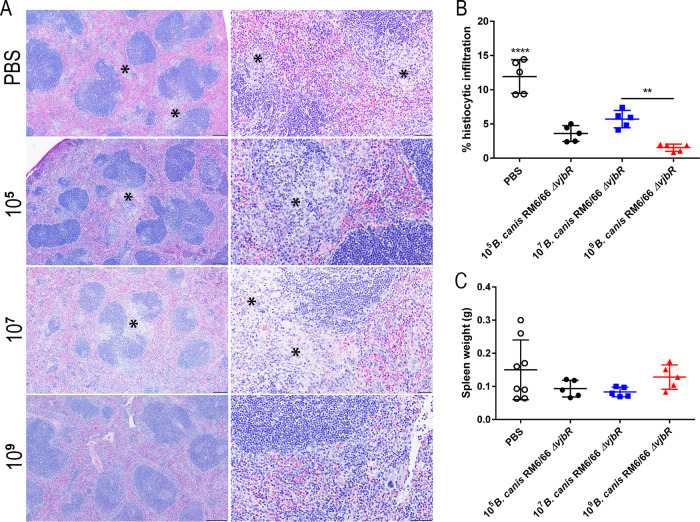
Vaccination with 10^9^ CFU of B. canis RM6/66 Δ*vjbR* protects mice against development of histiocytic inflammation in the spleen following challenge with B. canis. Female C57BL/6 mice were vaccinated i.p. with various doses of B. canis RM6/66 Δ*vjbR* or PBS, challenged i.p. at 4 weeks postvaccination with 10^7^ CFU of wild-type B. canis RM6/66, and euthanized at 1 and 2 weeks postchallenge. (A) H&E staining of the spleen at low magnification (left) and high magnification (right) in vaccinated mice at 2 weeks postchallenge. Notice multifocal foci of histiocytic inflammation (asterisks) within the red pulp and impinging on lymphoid follicles. Bars, 200 μm (left) and 50 μm (right). (B) Spleens at 2 weeks postchallenge were evaluated using QuPath Bioimage analysis v. 0.1.2. Foci of histiocytic inflammation were annotated and compared to the total area in microns squared with data presented as the total percentages of splenic area occupied by inflammation. (C) Splenic weight was calculated following challenge, with no significant differences noted between groups. Data are expressed as the means ± standard deviations. **, *P* < 0.01; ****, *P* < 0.0001 (Tukey’s multiple-comparison test).

Spleens were examined histologically following challenge and assessed for degree of histiocytic infiltration. Macrophages commonly formed distinct granulomas within the marginal zone and sinuses bordering lymphoid follicles in unvaccinated mice and in mice vaccinated with the lower doses of 10^5^ and 10^7^ CFU (Fig. 4A). These areas were annotated using QuPath software, and the total percentage of the spleen area occupied by histiocytic inflammation was calculated and compared between groups. At 2 weeks postchallenge, mice vaccinated with 10^9^ CFU of B. canis RM6/66 Δ*vjbR* showed significantly less histiocytic infiltration than the unvaccinated control group and mice vaccinated with 10^7^ CFU (Fig. 4B). In fact, foci of histiocytic inflammation in mice in the high-dose group were scarce, with the predominant histologic change being mild EMH. Despite higher levels of histiocytic inflammation in unvaccinated control mice, no significant differences in splenic weight were noted between vaccinated and unvaccinated mice (Fig. 4C).

### Subcutaneous vaccination provides significant protection against challenge.

Considering the reduction in organ colonization and the protection against splenic histiocytic inflammation elicited by a high dose of the vaccine, the experimental protocol was modified in an effort to improve the protective efficacy of B. canis RM6/66 Δ*vjbR* and to explore an alternative more practical vaccination route. Mice in the second protection study were all vaccinated with the high dose of 10^9^ CFU, with additional components added in an attempt to stimulate a stronger cell-mediated immune response. Some groups of mice received Quil-A, an adjuvant which was previously shown to provide higher levels of protection in mice against B. canis infection than other commonly used adjuvants, including Montanide and incomplete Freund’s adjuvant (IFA) (10). Subsets of mice received a lysate of the vaccine strain and/or a booster dose 2 weeks following the initial vaccination (see Fig. S3). Vaccinations were administered subcutaneously (s.q.) due to safety concerns associated with i.p. injection of Quil-A.

10.1128/mSphere.00172-20.3 FIG S3 Subcutaneous vaccination of mice with B. canis RM6/66 Δ*vjbR* protects against histiocytic inflammation in the liver following B. canis challenge. Female C57BL/6 mice were vaccinated with 10^9^ CFU of B. canis RM6/66 Δ*vjbR* s.q., with some mice receiving the adjuvant Quil-A, a lysate of the vaccine strain, and/or a booster vaccination 2 weeks following the first vaccination. One group of mice was vaccinated with PBS as a negative control. Mice were challenged at 8 weeks postvaccination with 10^7^ CFU of B. canis RM6/66 i.p., and livers were evaluated histopathologically at 2 weeks postchallenge. (A) H&E staining of the liver following challenge. Note the multifocal foci of histiocytic inflammation, or microgranulomas (arrows), within the red pulp, most numerous in mice in the first 5 groups. Bars, 100 μm. (B) The number of microgranulomas per liver section at 2 weeks postchallenge was counted and compared between groups. Data are expressed as the means ± standard deviations. **, *P* < 0.01; ***, *P* < 0.001; ****, *P* < 0.0001 (Tukey’s multiple-comparison test). Download FIG S3, PDF file, 0.4 MB. Copyright © 2020 Stranahan et al. 2020 Stranahan et al. This content is distributed under the terms of the Creative Commons Attribution 4.0 International license.

**FIG 5:**
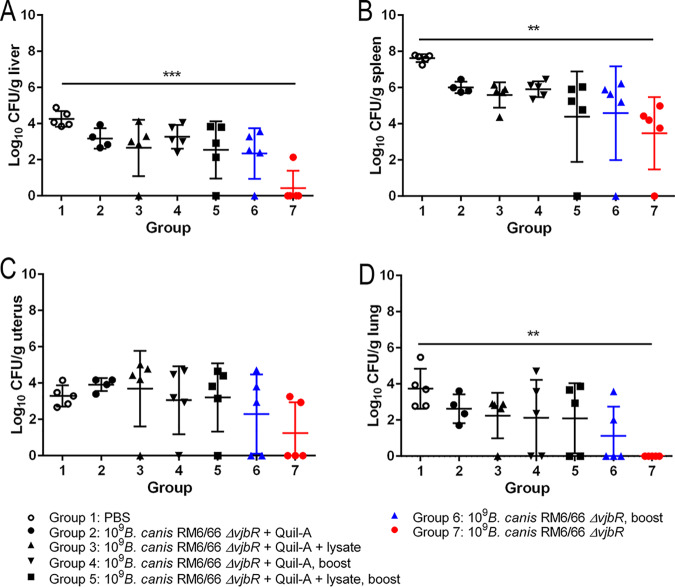
Subcutaneous vaccination of mice with B. canis RM6/66 Δ*vjbR* provides superior protection against organ colonization following B. canis RM6/66 challenge. Female C57BL/6 mice were vaccinated with 10^9^ CFU of B. canis RM6/66 Δ*vjbR* s.q., with some mice receiving the adjuvant Quil-A (15 μg), a lysate of the vaccine strain (50 μg), and/or a booster vaccination 2 weeks following the first vaccination. One group of mice was vaccinated with PBS as a negative control. Mice were challenged at 8 weeks postvaccination with 10^7^ CFU of B. canis RM6/66 i.p., and bacterial colonization was assessed at 2 weeks postchallenge in the liver (A), spleen (B), uterus (C), and lung (D). All data are expressed as the means ± standard deviations. **, *P* < 0.01; ***, *P* < 0.001 (Tukey’s multiple-comparison test).

Organ colonization was examined only at 2 weeks postchallenge owing to the lack of stark differences between 1 and 2 weeks postchallenge results following i.p. vaccination. Following challenge, mice vaccinated with a single dose of the vaccine s.q. exhibited significant reductions in organ colonization in the liver, spleen, and lung, with differences of 3.83 log, 4.143 log, and 3.74 log, respectively, compared to that in unvaccinated controls (Fig. 5). Four of the five mice in group 7 demonstrated widespread lack of detectable colonization, with 1 of these mice showing no detectable colonization in any examined organ. Although reduction in organ colonization was not significant, mice vaccinated with 10^9^ CFU B. canis RM6/66 Δ*vjbR* with a 2-week booster were also afforded high levels of protection, with reductions in colonization of 1.91 log in the liver, 3.03 log in the spleen, and 2.62 log in the lung. The level of organ colonization between mice vaccinated with a single dose (group 7) versus those that received a booster dose (group 6) was not significantly different. Unexpectedly, addition of the adjuvant, Quil-A, and/or a lysate of the vaccine strain did not improve protection against colonization. Rather, log reduction in organ colonization in these groups was inferior to that achieved by the vaccine alone, either in single dose or booster format (Fig. 5).

**FIG 6:**
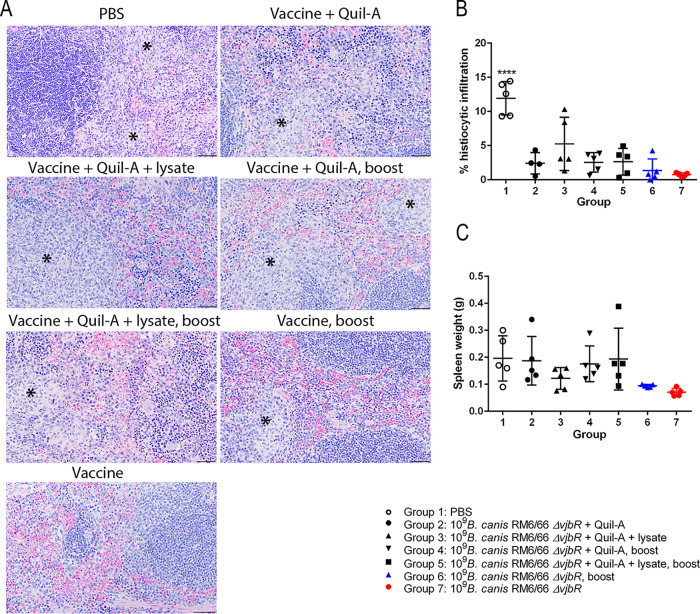
Subcutaneous vaccination of mice with B. canis RM6/66 Δ*vjbR* protects against inflammation in the spleen following challenge. Female C57BL/6 mice were vaccinated with 10^9^ CFU of B. canis Δ*vjbR* s.q., with some mice receiving the adjuvant Quil-A, a lysate of the vaccine strain, and/or a booster vaccination 2 weeks following the first vaccination. One group of mice was vaccinated with PBS as a negative control. Mice were challenged at 8 weeks postvaccination with 10^7^ CFU of B. canis RM6/66 i.p., and spleens were weighed and evaluated histopathologically at 2 weeks postchallenge. (A) H&E staining of the spleen following challenge. Note the multifocal foci of histiocytic inflammation (asterisks) within the red pulp, most numerous in mice in the first 5 groups. Bars, 50 μm. (B) Spleens at 2 weeks postchallenge were evaluated using QuPath Bioimage analysis v. 0.1.2. Foci of histiocytic inflammation were annotated and compared to the total area microns squared, with data presented as the total percentages of splenic area occupied by inflammation. (C) Splenic weight was calculated following challenge, with no significant differences noted between groups. All data are expressed as the means ± standard deviations. ****, *P* < 0.0001 (Tukey’s multiple-comparison test).

Histologic examination of the spleen following challenge revealed a significant reduction in the amount of histiocytic infiltration in all vaccinated mice (Fig. 6A and B). Although the change was not significant, mice vaccinated with 1 to 2 doses of B. canis RM6/66 Δ*vjbR* alone demonstrated the smallest amount of histiocytic inflammation, corresponding to the superior reduction in organ colonization. Despite higher levels of histiocytic inflammation in unvaccinated control mice, no significant differences in splenic weight were noted between vaccinated and unvaccinated mice (Fig. 6C). Subcutaneous vaccination with 10^9^ CFU of B. canis RM6/66 Δ*vjbR* also resulted in significantly fewer microgranulomas in the liver at 2 weeks postchallenge, with mice in group 6 (vaccine with booster) and group 7 (single vaccine dose) demonstrating the greatest reduction (Fig. S3).

### The B. canis RM6/66 Δ*vjbR* vaccine induces an immune response in mice and in canine cells.

**FIG 7:**
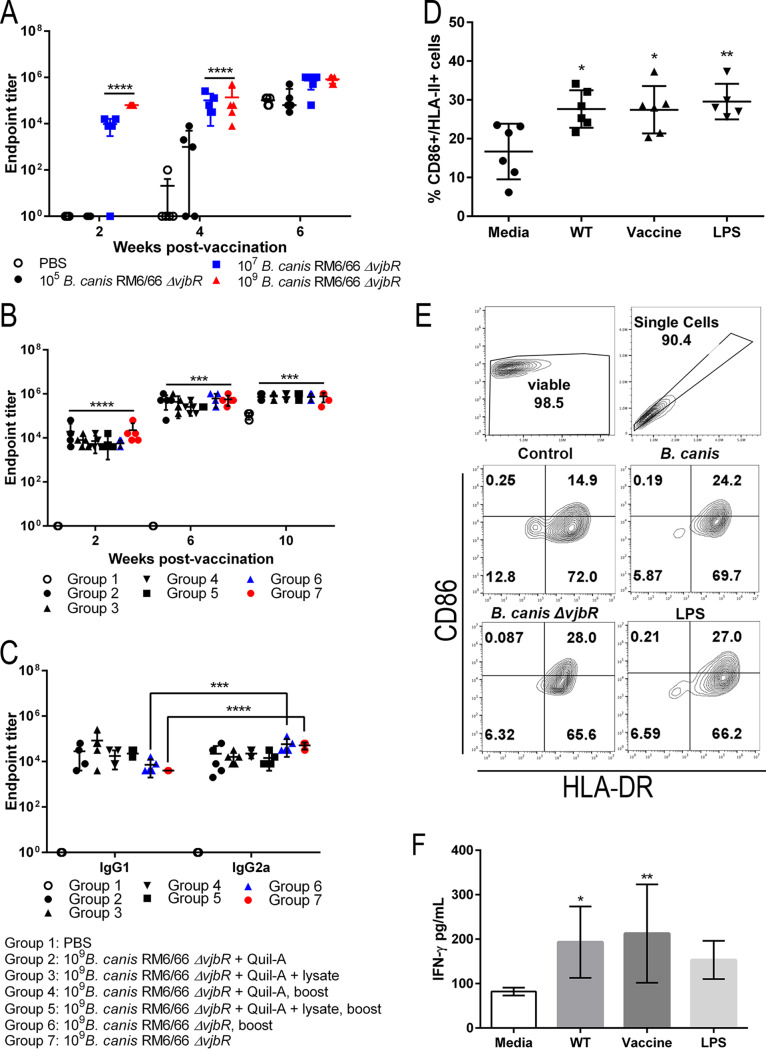
Vaccination with B. canis RM6/66 Δ*vjbR* stimulates a potent humoral immune response in mice and induces activation of canine dendritic cells *in vitro*. (A) Mice were vaccinated with various doses of B. canis RM6/66 Δ*vjbR* i.p. and challenged at 4 weeks postvaccination with 10^7^ CFU of B. canis RM6/66. Vaccination resulted in a significant increase in anti-*Brucella* IgG endpoint titers by 2 weeks postvaccination at 10^7^ and 10^9^ CFU. (B and C) Additional mice were vaccinated with 10^9^ CFU B. canis RM6/66 Δ*vjbR* s.q. with/without an adjuvant, lysate of the vaccine strain, or 2-week booster dose. Mice were challenged at 8 weeks postvaccination. All groups developed a significant increase in anti-*Brucella* IgG titers following vaccination (B), with significant increases in both IgG1 and IgG2a titers (C). Mice in groups 6 (two doses of vaccine) and 7 (single dose of vaccine) developed a greater rise in IgG2a than in IgG1 (C). (D) Exposure of canine dendritic cells *in vitro* to wild-type B. canis (WT), B. canis RM6/66 Δ*vjbR* (vaccine), or E. coli LPS resulted in a significant increase in double expression of the activation markers CD86 and HLA-DR. Percentages of positive cells were compared to FMO (fluorescence minus one) controls. (E) Representative flow cytometric images demonstrating expression of CD86 and HLA-DR. (F) Stimulation of canine dendritic cells with B. canis RM6/66 (WT) or B. canis RM6/66 Δ*vjbR* (vaccine) also induced a significant increase in IFN-γ secretion. All data are expressed as the means ± standard deviations. *, *P* < 0.05; **, *P* < 0.01; ***, *P* < 0.001; ****, *P* < 0.0001 (Tukey’s multiple-comparison test).

To understand the mechanism behind protection against organ colonization and development of histopathologic lesions in vaccinated mice, the humoral immune response to vaccination and challenge was assessed in all groups. Vaccination by both i.p. and s.q. routes resulted in a humoral immune response in mice, as indicated by a rise in anti-*Brucella* total IgG. The increase in total IgG subsequent to i.p. vaccination followed a dose-dependent pattern (Fig. 7A). Mice vaccinated with the higher doses of 10^7^ and 10^9^ CFU of B. canis RM6/66 Δ*vjbR* exhibited significantly higher IgG titers by 2 weeks postvaccination than control animals and those vaccinated with 10^5^ CFU. In contrast, mice vaccinated with the low dose of 10^5^ CFU did not develop a notable antibody response to vaccination alone and only demonstrated a significant increase in IgG following challenge, as seen with the unvaccinated control group. Antibody titers increased following challenge with the two higher vaccine doses (10^7^ and 10^9^ CFU), although the change was not significant (Fig. 7A). Subcutaneous vaccination with all formulations resulted in an increase in anti-*Brucella* IgG at 2 weeks, with titers peaking at 6 weeks postvaccination (Fig. 7B). As with i.p. vaccination, titers increased following challenge, although not significantly.

To further investigate the mechanism behind vaccination-induced protection, the levels of anti-*Brucella* IgG1 and IgG2a in vaccinated mice were analyzed. At the peak of the humoral immune response at 6 weeks postvaccination, vaccination in all groups resulted in a significant increase in IgG1 and IgG2a compared to that in unvaccinated controls (Fig. 7C). Interestingly, IgG2a levels, which signal a cell-mediated, or TH1, immune response, were significantly higher than IgG1 only for groups 6 and 7 (*P* < 0.001) which received two doses and a single dose of B. canis RM6/66 Δ*vjbR*, respectively. Further demonstrating the TH1 skew to the immune response in groups 6 and 7 are the low IgG1/IgG2a optical density (OD) ratios of 0.098 and 0.041, respectively, compared to ratios of up to 2.06 for group 2.

Following assessment of the immune response in vaccinated mice, the potential for B. canis RM6/66 Δ*vjbR* to induce a cell-mediated immune response in the natural canine host was investigated *in vitro*. Dendritic cells were selected as they are key antigen-presenting cells that play a critical role in the initial immune response to foreign antigens, including those associated with vaccines. Stimulation of mononuclear derived dendritic cells from healthy dogs with both wild-type B. canis RM6/66 and B. canis RM6/66 Δ*vjbR* resulted in a significantly higher percentage of cells expressing both HLA-DR, an MHC class II surface receptor, and CD86, a costimulatory protein for T lymphocytes, changes which were also observed in cells stimulated with the Escherichia coli lipopolysaccharide (LPS) positive control (Fig. 7D and E). Additionally, secretion of gamma interferon (IFN-γ) by the dendritic cells was evaluated, as this cytokine plays critical roles in the development of a cell-mediated immune response. Stimulation with both wild-type B. canis RM6/66 and B. canis RM6/66 Δ*vjbR* resulted in significantly higher production of IFN-γ (Fig. 7F).

## DISCUSSION

Canine brucellosis, primarily caused by Brucella canis, remains an endemic disease in multiple regions, including the southern United States. While considered significantly less virulent for humans than the smooth *Brucella* strains, B. canis is zoonotic and thus a public health concern (3, 26, 27). While numerous factors complicate the control of canine brucellosis, the lack of a protective vaccine for use in dogs is perhaps the most significant. Live attenuated vaccines (LAVs) are considered to produce the highest levels of protection owing to their ability to mimic the wild-type organism in terms of cell invasion and tissue tropism (16, 27). However, due to concerns for safety and risk for reversion to virulence, the majority of brucellosis vaccine research in recent years has focused on investigating killed vaccines (28). Few studies into candidate vaccines for B. canis have been conducted, with LAV candidates inducing superior or comparable levels of protection compared to those of killed vaccines (10–15). In this study, we chose to investigate an LAV vaccine candidate for canine brucellosis, B. canis RM6/66 Δ*vjbR*.

To be safe for use in animals, a LAV must be attenuated and persist long enough in the host to induce a lasting protective immune response. This study demonstrated that the LAV candidate, B. canis RM6/66 Δ*vjbR*, was significantly attenuated both *in vitro* in canine cells and in mice, with clearance in all organs achieved at 3 weeks postvaccination. While yet to be tested in the natural canine host, this provides evidence toward vaccine safety.

Protective efficacy was first investigated following the frequently utilized i.p. route of vaccination. While mice vaccinated with 10^9^ CFU of B. canis RM6/66 Δ*vjbR* showed significant protection against colonization, bacterial levels in the spleen at 2 weeks postchallenge remained at approximately 4 log. To improve protective efficacy, alternative strategies involving route, use of adjuvant, and booster doses were investigated. Importantly, subcutaneous vaccination resulted in comparable levels of protection. Recently, investigations into different infection routes for smooth *Brucella* spp. in mice have demonstrated that *Brucella* disseminates almost immediately to the spleen following both i.p. and intradermal infections, with spread from the skin largely dependent on dendritic cells (29). These data and those in the present study support the advantage of the s.q. route of vaccination over the i.p. route, as it is likely to provide at least comparable protection and represents a far more feasible vaccination approach. Additionally, no significant differences were observed in protection in mice that received 1 versus 2 vaccine doses, an encouraging finding, as protection triggered by a single vaccination is the most important factor influencing the use of veterinary vaccines by marginalized populations to which canine brucellosis represents the most significant threat (30).

Typically, vaccine-induced protection against *Brucella* species infection in the mouse model is restricted to evaluation of splenic colonization, with few studies investigating the liver and, even less commonly, the lung (31–33). In this study, s.q. vaccination with B. canis RM6/66 Δ*vjbR* protected against colonization not only in the liver and spleen, known target organs for *Brucella* spp., but also in the lung, evidence toward a more systemic level of protection. Despite the fact that reproductive disease is a major manifestation of brucellosis and induction of abortion is a significant concern for LAVs, colonization of the uterus is rarely assessed in vaccine efficacy studies in laboratory animal models (34). This is despite the fact that *Brucella* spp. can colonize both the pregnant and nonpregnant uteri of mice and guinea pigs (24, 35, 36). Though not significant, s.q. vaccination resulted in a notable reduction in uterine colonization (Fig. 5). The significance of uterine protection in mice in the context of B. canis infection is uncertain, and protection in pregnant mice remains to be investigated.

Protection against colonization correlated well with protection against the development of histiocytic inflammation in the spleen and liver. In this study, vaccination with 10^9^ CFU of B. canis RM6/66 Δ*vjbR* resulted in transient splenomegaly, a gross change that is typically associated with disease in brucellosis. However, histopathologic examination revealed the presence of extramedullary hematopoiesis (EMH), or production of blood cells outside the bone marrow in response to increased demand (37). In the absence of significant inflammatory lesions in vaccinated mice, the transient EMH likely represents a strong immune response in reaction to the vaccine. Such a change has been reported in other vaccine studies in mice, with evidence that vaccines which induce EMH result in superior levels of protection, likely by providing stores of immature immune cells primed for response upon subsequent infection (38). This not only provides further support for the protective efficacy of B. canis RM6/66 Δ*vjbR* but also highlights the importance of histopathology in appropriately interpreting gross changes in vaccine studies.

To begin deciphering the underlying immune mechanism of protection induced by B. canis RM6/66 Δ*vjbR* in mice, the humoral immune response to vaccination was investigated. It is well established that control of smooth *Brucella* species infection is reliant on a strong TH1 immune response and, although unconfirmed, is suspected to play an important role in control of B. canis infection (17, 39). One component of this response is production of IgG2a antibodies which aid in opsonization of bacteria (17). In this study, the groups which received either 1 or 2 subcutaneous doses of B. canis RM6/66 Δ*vjbR* achieved both the greatest level of protection and significantly higher IgG2a titers than IgG1 titers. Interestingly, addition of an adjuvant Quil-A did not result in significant protection against organ colonization. Quil-A, widely used in veterinary vaccines, induces both a strong TH1 and TH2 response (40). Groups which received Quil-A showed no significant differences in IgG1 versus IgG2a titers, while groups that received the vaccine alone showed a TH1-skewed response. This even balance between a TH1- and TH2-biased rather than a TH1-skewed response could explain the differences in protection.

With evidence for the ability of B. canis RM6/66 Δ*vjbR* to protect against challenge in mice, its potential to activate immune cells in the natural canine host was investigated. Exposure of canine dendritic cells to wild-type B. canis RM6/66 and B. canis RM6/66 Δ*vjbR* resulted in increased double expression of the activation markers, HLA-DR and CD86, as well as increased secretion of IFN-γ. These changes represent activation of dendritic cells, critical antigen-presenting cells involved in development of a cell-mediated immune response (41). Interestingly, such activation changes were previously described in canine dendritic cells infected with wild-type B. canis (42). Investigation of dendritic cell activation is an increasingly common component in vaccine studies, with evidence that enhanced dendritic cell activation may correlate with superior protection (43–45). Activation of canine dendritic cells by B. canis RM6/66 Δ*vjbR* is intriguing, although protective efficacy in dogs remains to be investigated.

The attenuation and efficacy of B. canis RM6/66 Δ*vjbR* compares well to that noted with other *vjbR* deletion mutants developed in our lab (20–23). A recent study also investigated a *vjbR* deletion mutant of B. canis and noted significant levels of protection induced by i.p. vaccination with 10^7^ CFU, lower than the dose of 10^9^ CFU required to achieve protection in this study (13). This discrepancy may be related to a difference in the mouse strain utilized (BALB/c versus C57BL/6) or the size of the deletion, as 753 bp of the *vjbR* gene was removed in our study compared to 201 bp.

This work demonstrates that the vaccine candidate B. canis RM6/66 Δ*vjbR* provides significant protection against organ colonization and histopathologic lesions in mice and induces a TH1-skewed humoral immune response. Additionally, B. canis RM6/66 Δ*vjbR* is capable of activating canine dendritic cells. We also provide evidence that the subcutaneous vaccination route provides comparable, if not superior, protection levels to those with i.p. vaccination, and this more practical method should be explored in future vaccine studies. The C57BL/6 mouse model utilizing a challenge dose of 10^7^ CFU serves well for investigating B. canis vaccine candidates, and it is critical that a standard approach in mice such as this be adopted for accurate comparison of vaccine candidates. Further studies to improve efficacy, such as the use of microencapsulation or other slow-release modalities, in addition to investigation of safety and protection in the natural canine host may be undertaken. Overall, these results suggest that B. canis RM6/66 Δ*vjbR* could serve as a safe and effective vaccine for protection against canine brucellosis.

## MATERIALS AND METHODS

### Ethics statement.

Animal experiments were conducted in an approved facility in strict accordance with all university and federal regulations. Mouse experimentation (protocol 2018-0046) and canine blood collection protocols (protocol 2018-0457 CA) were reviewed and approved by the Texas A&M University Laboratory Animal Care and Use Committee. All protocols were approved and were in accordance with the Institutional Animal Care and use Committee (IACUC) policies of Texas A&M University. Texas A&M is accredited by the Association for the Assessment and Accreditation of Laboratory Animal Care, International (AAALAC).

### Animals.

Female C57BL/6J (6 to 8 weeks old) were obtained from the Texas A&M Institute for Genomic Medicine and housed in microisolator caging in biosafety level 2 and 3 facilities at Texas A&M College of Veterinary Medicine. All mice were acclimated to the facility for 5 days prior to vaccination or infection and were maintained on a 12-h/12-h light-dark cycle with *ad libitum* access to food and filtered water. Mice were monitored daily for signs of pain or distress according to the guidelines of the Animal Research Advisory Committee published by the National Institutes of Health. For generation of dendritic cells *in vitro*, fresh blood was collected from healthy client-owned dogs presenting to Texas A&M University Small Animal Hospital following informed client consent. All dogs were up to date on routine vaccinations (canine distemper virus, canine parvovirus, canine adenovirus, parainfluenza virus, and rabies virus) and had no systemic inflammatory, infectious, or neoplastic conditions. Blood (20 to 25 ml) was collected by licensed veterinary technicians from nonanesthetized dogs via the saphenous vein.

### Bacterial strain.

B. canis ATCC RM6/66 was used for these studies. Bacterial stocks were stored at −80°C in 10% glycerol and were routinely grown on tryptic soy agar (TSA) plates or in standard tryptic soy broth (TSB). Bacteria were harvested from plates using phosphate-buffered saline (PBS), pH 7.2 (Gibco), and adjusted to a final concentration of either 10^5^, 10^7^, or 10^9^ CFU/0.1 ml using a Klett colorimeter meter reading against a standard curve. Viable counts were retrospectively confirmed by serial dilution and plating onto TSA plates.

### Generation of B. canis RM6/66 Δ*vjbR* deletion mutant.

Brucella canis RM6/66 Δ*vjbR* was generated using the kanamycin-resistant pUC19 suicide plasmid and the ampicillin-resistant pCVD442 plasmid. Briefly, a 650-bp upstream fragment and a 350-bp downstream fragment of the *vjbR* gene (BCAN_RS10670) were amplified. The upstream fragment was amplified using forward primer (CCCCCGAGCTCTTATCGGCCAGTTGGAAAAG) and reverse primer (CCCCCGGATCCGAGATCAAGACTCATTGGAAATATCC). The downstream fragment was amplified using forward primer (CCCCCGGATCCCATCTCGTCTGATCAACATGG) and reverse primer (CCCCCTCTAGAATTTCTATCCCGGCACACTG). A BamHI restriction site was placed at the 5′ end and an Xbal restriction at the 3′ end for both PCR products. Following PCR amplification and restriction digestion, upstream and downstream gene segments were separately cloned into pUC19 plasmids. Upstream and downstream gene segments were joined using crisscross reactions, and positive clones were selected, with proper orientation confirmed by PCR amplification and sequencing. E. coli BL21 cells were used to amplify the plasmid, and transformed clones were selected based on kanamycin resistance.

To remove antibiotic resistance, the *vjbR* deletion cassette from the pUC19 plasmid was restriction digested and ligated into the pCVD442 plasmid. Ligated products were transformed into E. coli β2155 using heat shock, and successful transformation was confirmed following sequencing. The pCVD442 plasmid was transformed into B. canis RM6/66 via electroporation. Positive clones were selected based on ampicillin susceptibility (100 μg/ml) and sucrose resistance (10%). Successful deletion of the *vjbR* gene (753 bp) was confirmed using PCR amplification and sequencing.

### Cellular infection assays.

Cellular infections for estimation of bacterial invasion and replication were performed using canine DH82 macrophage-like cells (ATCC CRL-10389). Cells were grown to confluence in 24-well tissue culture plates using Eagle’s minimum essential medium (EMEM) plus 10% fetal bovine serum. B. canis RM6/66 and B. canis RM6/66 Δ*vjbR* were grown on TSA plates for 3 days and then subcultured in 5 ml of TSB in 50-ml, sterile conical plastic tubes. Liquid cultures were maintained at 37°C and 200 rpm for 16 to 20 h. Cells were infected with a multiplicity of infection (MOI) of 100, and all inocula were serially diluted and plated on TSA to confirm inoculation dose. Plates containing infected cells were centrifuged at 1,000 rpm at 22°C, incubated for 30 min at 37°C and 5% CO_2_, and washed with warm culture medium. Extracellular bacteria were eliminated by addition of 50 μg/ml of gentamicin for 1 h. After 1, 24, and 48 h of incubation, plates were washed with phosphate-buffered saline (PBS), and cells were lysed by treatment with 0.5% Tween 20 for 5 min with vigorous scraping. Aliquots were serially diluted, plated on TSA, and incubated at 37°C for 3 days for CFU determination. For determination of cell death, lactate dehydrogenase (LDH) released into cell culture supernatants of infected and uninfected cells was determined at all time points using the CytoTox 96 nonradioactive cytotoxicity assay kit (G1780; Promega) according to the manufacturer’s instructions. Cell cytotoxicity was expressed as the percentage of LDH release, which was calculated using the following formula: percentage of LDH release = 100 × (test LDS release − spontaneous release)/(maximum release − spontaneous release).

### Safety and protection studies in mice.

For all experiments, mice were randomly divided into groups (*n *= 5). For assessment of virulence and kinetics of organ colonization of the vaccine strain, mice were vaccinated intraperitoneally (i.p.) with either PBS or 10^5^, 10^7^, or 10^9^ CFU of B. canis RM6/66 Δ*vjbR*. Mice were sacrificed by CO_2_ asphyxiation and cervical dislocation at weekly intervals for 4 weeks. For dose titration and preliminary investigation of protective efficacy, mice vaccinated i.p. with the aforementioned doses of B. canis RM6/66 Δ*vjbR* were challenged i.p. with 10^7^ CFU of wild-type B. canis RM6/66 in 100 μl of PBS at 4 weeks postvaccination and euthanized at 1 and 2 weeks postchallenge.

In a second protection study, mice were vaccinated subcutaneously (s.q.) with 100 μl PBS containing 10^9^ CFU of B. canis RM6/66 Δ*vjbR*. Some groups additionally received 15 μg of Quil-A and/or 50 μg of a heat-killed lysate of the vaccine strain either once or twice (days 0 and 14). Lysates were prepared following 3 passages through a French press. The components of the vaccine per group and dosing scheduled are highlighted in Fig. S4 in the supplemental material. Quil-A (Brenntag Biosector, Denmark) was prepared according to the manufacturer’s instructions. Animals were examined by a veterinarian to evaluate general health status and local adverse reactions at the injection site. Eight weeks after the initial vaccination, all mice were challenged with 10^7^ CFU of B. canis RM6/66 by i.p. inoculation, with euthanasia following 2 weeks later.

10.1128/mSphere.00172-20.4 FIG S4 Schematic of experimental design and vaccine composition by group. Female C57BL/6 mice (*n* = 5) were vaccinated subcutaneously with 10^9^ CFU B. canis RM6/66 Δ*vjbR*, with some mice receiving additional components of the adjuvant Quil-A (15 μg) or a lysate of the vaccine strain (50 μg). Three groups of mice received a booster vaccination 2 weeks following the first vaccination. Blood was collected from the tail vein every 2 weeks until 10 weeks postvaccination. Mice were challenged i.p. with 10^7^ CFU of B. canis RM6/66 and euthanized 2 weeks postchallenge. Download FIG S4, TIF file, 0.2 MB. Copyright © 2020 Stranahan et al. 2020 Stranahan et al. This content is distributed under the terms of the Creative Commons Attribution 4.0 International license.

For all animal experiments and at each time point, samples of liver, spleen, uterus, and lung were aseptically collected in 1 ml PBS, homogenized, and serially diluted, and 100 μl of each dilution was plated onto Farrell’s medium (TSA plus Brucella 391 Oxoid supplement, equine serum, and 50% dextrose) and incubated at 37°C. Bacterial colonies were enumerated after 72 h to quantify tissue colonization. Levels of infection were expressed as mean values and standard deviations (*n* = 5) of the log number of CFU per gram of tissue. Spleens were weighed at necropsy, and the aforementioned tissues, in addition to mesenteric lymph nodes and heart, were collected at each time point and fixed in 10% neutral buffered formalin for routine histopathologic evaluation. Tissues were routinely processed and embedded, sectioned at 4 μm, and stained with hematoxylin and eosin. Sections from the spleen following challenge were graded for severity of granulomatous inflammation using QuPath Bioimage analysis v. 0.1.2 (Belfast, Northern Ireland, UK) (46). Foci of granulomatous inflammation were annotated, and the percentage of total tissue area affected was calculated.

### Measurement of humoral immune response.

Mice were bled by ventral tail vein puncture prior to the start of each experiment, at each euthanasia time point, and at biweekly intervals following s.q. vaccination. Blood was centrifuged at 3,000 rpm for 5 min, and serum was collected for anti-B. canis-specific immunoglobulin G (IgG) indirect enzyme-linked immunosorbent assay (iELISA). Briefly, 96-well plates (Costar, Corning, NY, USA) were coated with 250 ng/well of B. canis RM6/66 heat-killed lysate in coating buffer (pH 9.6, 0.05 M carbonate buffer) at 4°C overnight. Plates were washed three times with PBS containing 0.05% Tween 20 (PBST), and nonspecific binding was blocked with 100 μl of 3% skim milk in PBST at room temperature for 2 h. Following three washes, 2-fold dilutions of sera in PBST containing 1% skim milk were added and incubated at 37°C for 1 h. Plates were washed five times, and horseradish peroxidase (HRP)-labeled goat anti-mouse IgG (1:2,000; Vector Laboratories), HRP-labeled rat anti-mouse IgG2a (1:2,000; Southern Biotech), or rat anti-mouse IgG1 (1:2000, clone SB77e; Southern Biotech) was added, followed by incubation at 37°C for 1 h. Afterwards, OPD peroxidase substrate (Sigma-Aldrich) was added (100 μl/well) and incubated for 30 min at 37°C in the dark. The enzyme reaction was stopped by addition of 0.5 M NaOH, and absorbance was measured at 450 nm. Endpoint titers were reported as the Log_10_ of the highest dilution giving an OD reading higher than the mean plus 2 standard deviations of that for the baseline sera. All assays were performed in triplicates, and the results are presented as the mean reciprocal endpoint titer.

### Isolation and infection of canine dendritic cells.

Canine dendritic cells (DCs) were derived as previously described (42, 47). Briefly, 20 to 25 ml of fresh heparinized blood was obtained from healthy dogs and diluted at a 1:1 ratio in PBS followed by gentle layering onto Histopaque, density 1.077 g/ml (Sigma-Aldrich), in 50-ml conical tubes at a blood/Histopaque ratio of 2:1. Gradients were centrifuged for 40 min at 700 × *g* at room temperature with no brake. Afterwards, the interface layer consisting of peripheral blood mononuclear cells (PBMCs) was collected and transferred to a new tube. Cells were incubated with red blood cell lysis buffer (Sigma-Aldrich) for 5 min at room temperature, followed by dilution in PBS and centrifugation at 500 × *g* for 5 min at 4°C. The cell pellet was washed twice in PBS, with the pellet finally being resuspended in separation buffer (PBS plus 0.5% bovine serum albumin [BSA] plus 2 mM EDTA, pH 7.2) for magnetic bead purification. CD14^+^ monocytes were isolated from the PBMCs using anti-human CD14 monoclonal antibody (clone TÜK4) conjugated to magnetic beads (MACS; Miltenyi Biotec, Bergisch Gladbach, Germany) according to the manufacturer’s instructions. Cells were counted, resuspended in complete medium (RPMI 1640 plus 10% fetal bovine serum [FBS] plus l-glutamine plus 10 U/ml penicillin plus 10 μg/ml streptomycin), and plated at a density of 5 × 10^5^ to 1 × 10^6^ cells/ml in 24-well plates. Culture medium was supplemented with 50 ng/ml of recombinant canine granulocyte-macrophage colony-stimulating factor (GM-CSF) (R&D Systems) and 30 ng/ml of recombinant canine interleukin 4 (IL-4) (R&D Systems) to induce differentiation to DCs. Half of the medium was replaced every 2 days, and monocytes were allowed to differentiate into DCs at 37°C and 5% CO_2_ for 7 days.

After 7 days in culture, canine DCs were infected at an MOI of 100 with B. canis RM6/66, B. canis RM6/66 Δ*vjbR*, PBS as a negative control, or 1 μg/ml LPS from Escherichia coli strain 0128:B12 (Sigma-Aldrich) as a positive control. Infected cells were incubated at 37°C and 5% CO_2_ for 1 h, followed by a 1-h treatment with 50 μg/ml gentamicin. Cultures were maintained for 24 h, and cells were collected into microcentrifuge tubes by gentle pipetting and washed twice with PBSA (PBS plus 0.5% BSA). Following centrifugation, supernatants were collected and stored at −20°C until further use. Afterwards, cells were incubated with 20 μl of monoclonal antibodies, mouse anti-human CD86-fluorescein isothiocyanate (FITC) (clone BU63; Bio-Rad) and mouse anti-human HLA-DRII-allophycocyanin (APC) (clone G46-6; BD Biosciences), per 100-μl reaction mixture of cells in PBSA for 30 min at 4°C in the dark. Cells were washed twice with PBSA and resuspended in 200 μl of 4% formaldehyde for 30 min at room temperature with addition of 10 μl of propidium iodide. Surface marker expression was assessed via flow cytometry using an BD Accuri C6 Plus flow cytometer (BD Biosciences). The amount of canine interferon γ in cell supernatants was measured via a commercial ELISA kit according to the manufacturer’s instructions (R&D Systems).

### Statistical analyses.

Analysis was performed using GraphPad Prism software, version 6.0 (GraphPad Software, San Diego, CA). The CFU data were normalized by log transformation and evaluated by two-way analysis of variance (ANOVA) repeated-measures test. Tukey’s multiple-comparison test was used to generate *P* values for mean comparisons. Splenic weight and histologic scores were compared using one-way ANOVA, and Tukey’s multiple-comparison test was used to generate *P* values. Canine dendritic cell samples were evaluated using FlowJo software, with absorbance gated for viability. Data were analyzed using fluorescence minus one (FMO) controls and were expressed as the mean ± standard deviation (SD) percentage of positive cells for each surface marker. Data were compared using one-way ANOVA, and Tukey’s multiple-comparison test was used to generate *P* values. In all analyses, a *P* value of less than 0.05 constituted statistical significance.
