# Supplementary figures and images for: Evaluation of the Efficacy of the Brucella canis RM6/66 ΔvjbR Vaccine Candidate for Protection against B. canis Infection in Mice
Source: mSphere. 2020 May 20;5(3):e00172-20. doi: 10.1128/mSphere.00172-20 (PMC7380573; doi:10.1128/mSphere.00172-20)

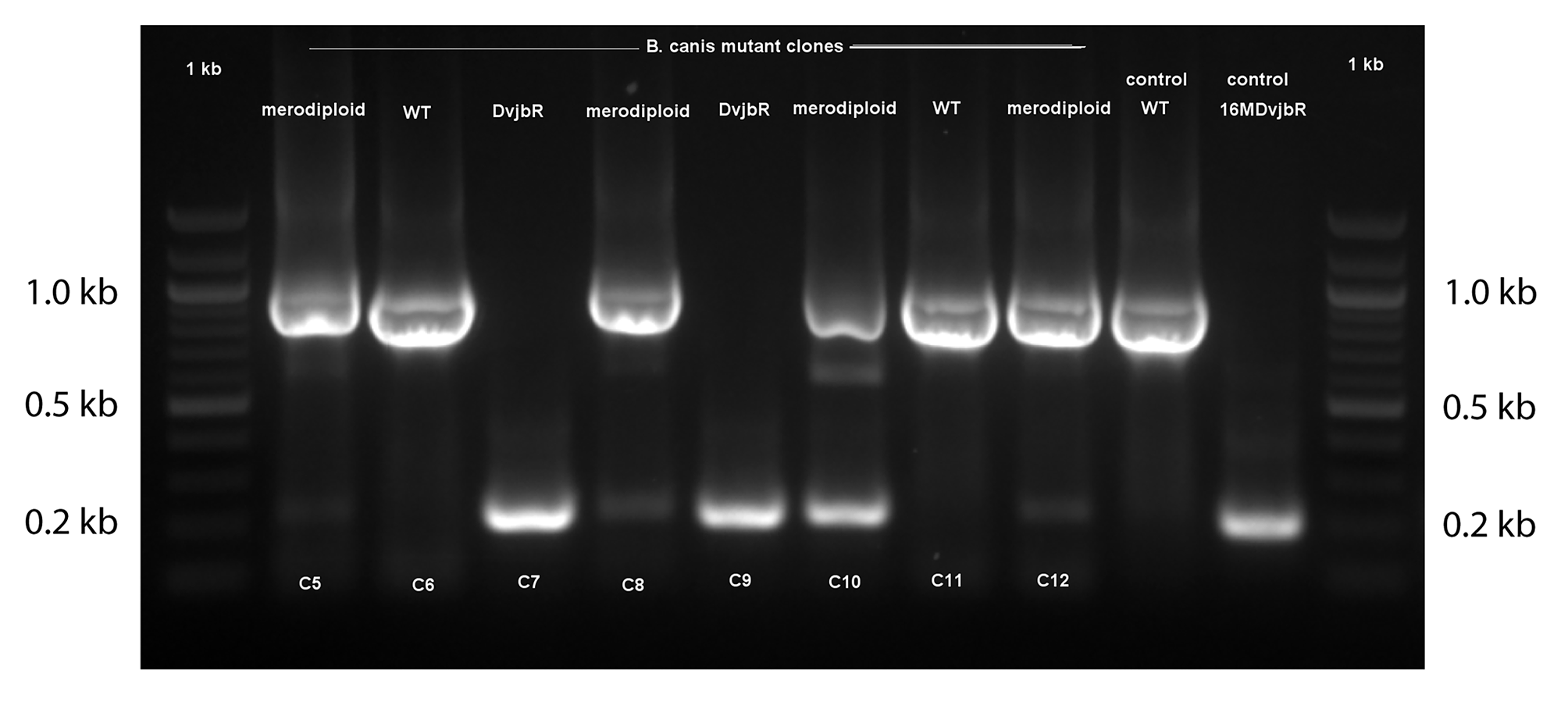

Supplement: FIG S1 [file mSphere.00172-20-sf001.tif]

PBS

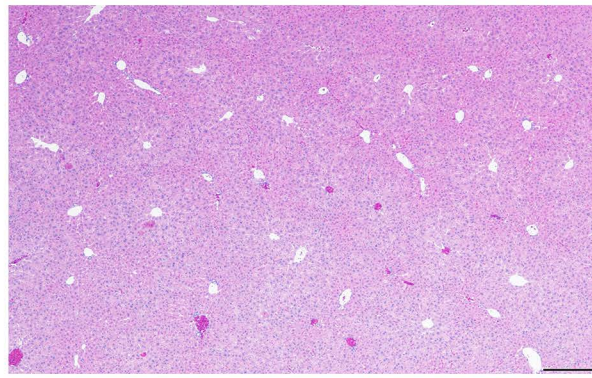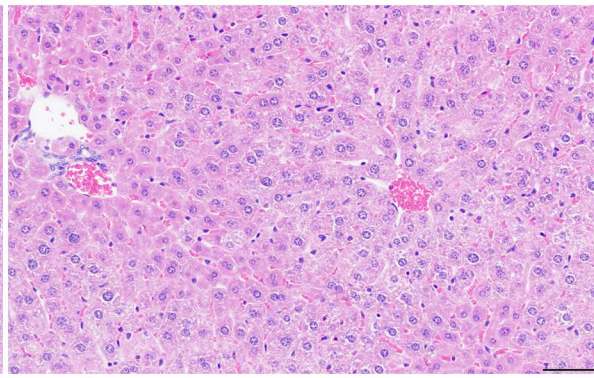

$10^5$

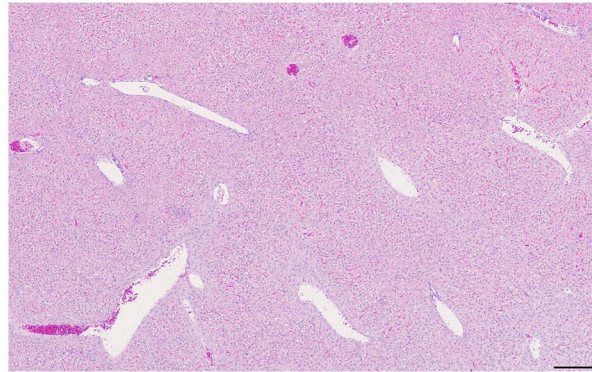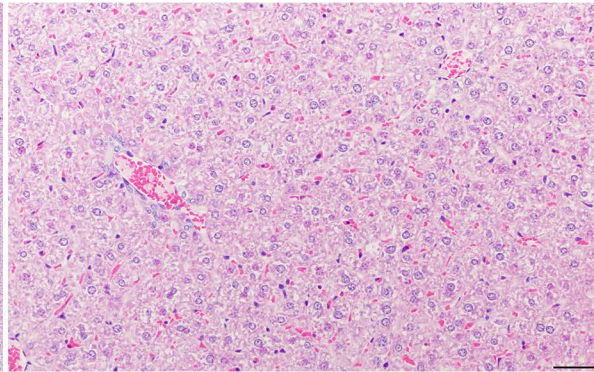

$10^7$

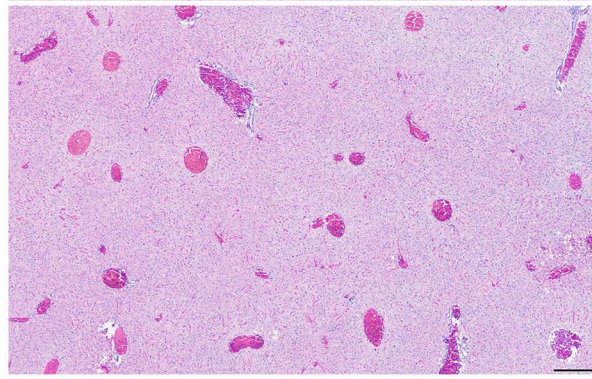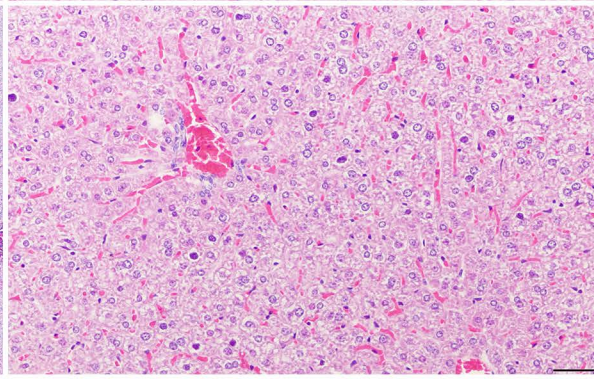

$10^9$

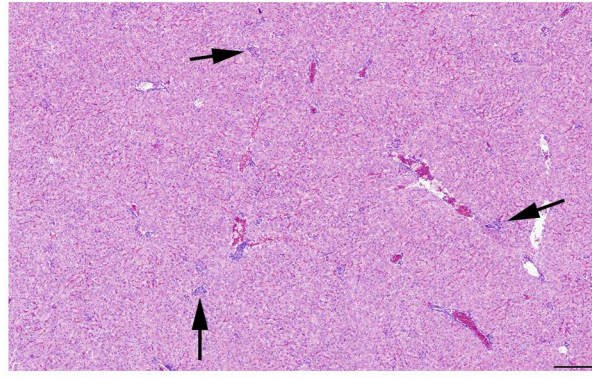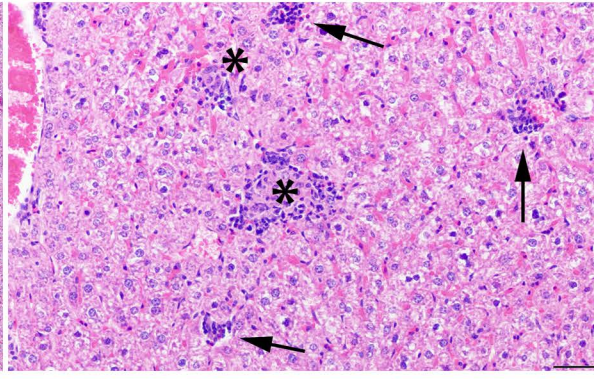

Supplement: FIG S2 [file mSphere.00172-20-sf002.pdf]

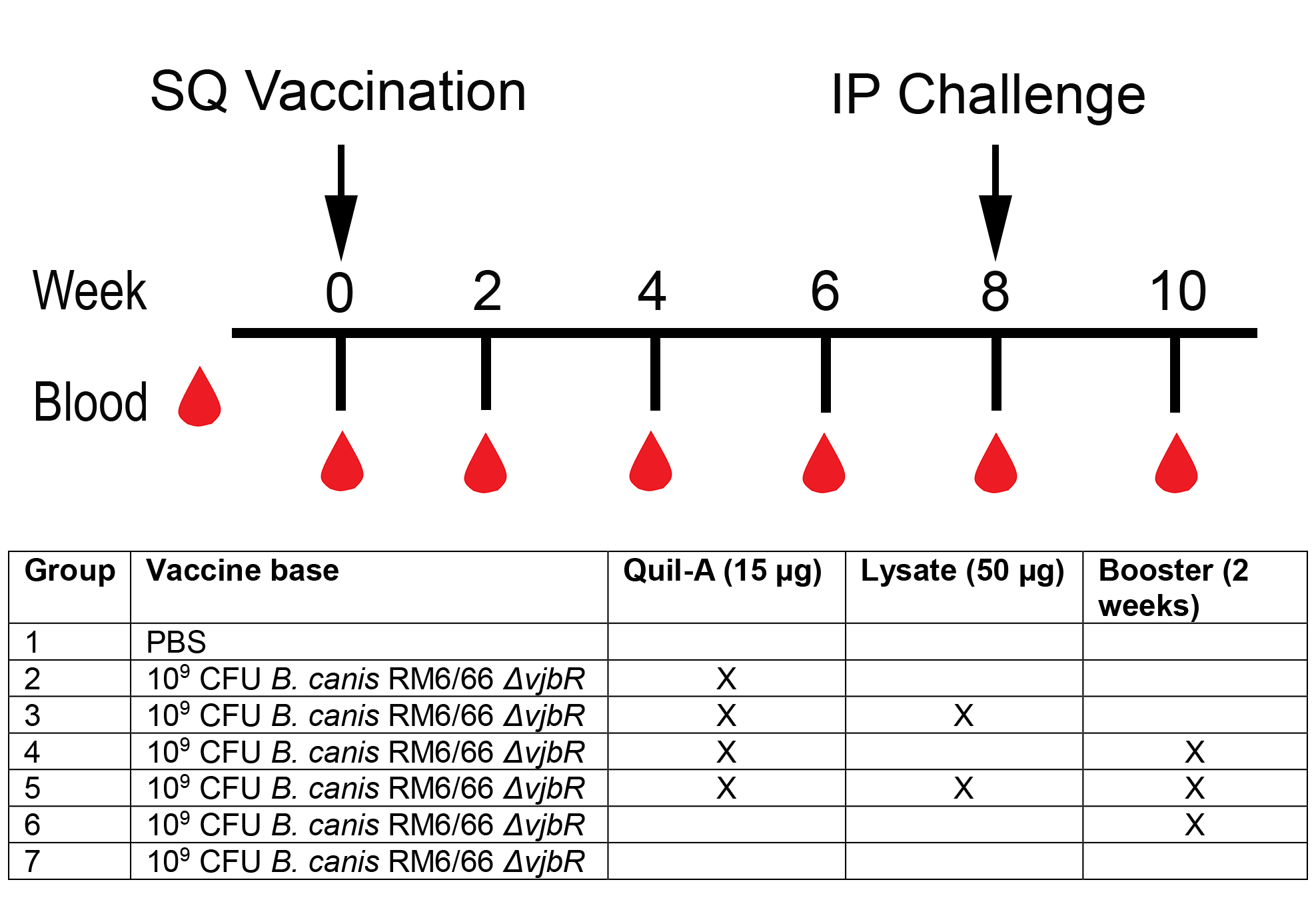

Supplement: FIG S4 [file mSphere.00172-20-sf004.tif]
