# Supplementary material for: Evaluation of the Efficacy of the Brucella canis RM6/66 ΔvjbR Vaccine Candidate for Protection against B. canis Infection in Mice
Source: mSphere. 2020 May 20;5(3):e00172-20. doi: 10.1128/mSphere.00172-20 (PMC7380573; doi:10.1128/mSphere.00172-20)
Supplement: FIG S3 [file mSphere.00172-20-sf003.pdf]

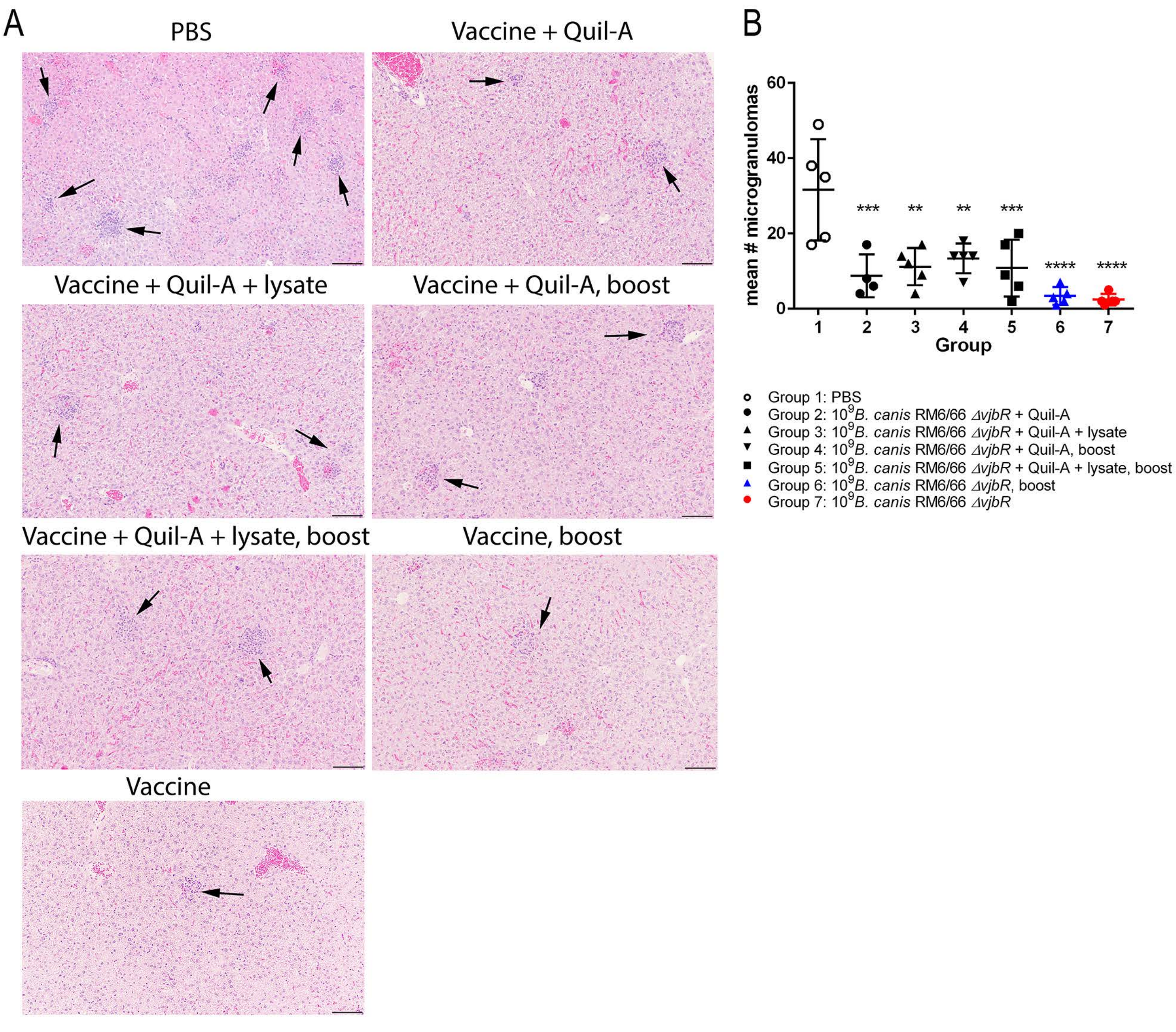
A
PBS
Vaccine + Quil-A
Vaccine + Quil-A + lysate
Vaccine + Quil-A, boost
Vaccine + Quil-A + lysate, boost
Vaccine, boost
Vaccine
B
mean # microgranulomas
60
40
20
0
***
**
**
***
****
****
1
2
3
4
5
6
7
Group
Group 1: PBS
Group 2: 10^{9} *B. canis* RM6/66 *ΔvjbR* + Quil-A
Group 3: 10^{9} *B. canis* RM6/66 *ΔvjbR* + Quil-A + lysate
Group 4: 10^{9} *B. canis* RM6/66 *ΔvjbR* + Quil-A, boost
Group 5: 10^{9} *B. canis* RM6/66 *ΔvjbR* + Quil-A + lysate, boost
Group 6: 10^{9} *B. canis* RM6/66 *ΔvjbR*, boost
Group 7: 10^{9} *B. canis* RM6/66 *ΔvjbR*
